# Retrospective analysis of outcome and toxicity after postoperative radiotherapy in patients with squamous cell carcinoma of the lip

**DOI:** 10.1177/0300891621996805

**Published:** 2021-03-01

**Authors:** Kristin Lang, Sati Akbaba, Thomas Held, Rami El Shafie, Benjamin Farnia, Nina Bougatf, Denise Bernhardt, Christian Freudlsperger, Peter K. Plinkert, Stefan Rieken, Jürgen Debus, Sebastian Adeberg

**Affiliations:** 1 Department of Radiation Oncology, University Hospital of Heidelberg, Heidelberg, Germany; 2 Heidelberg Institute of Radiation Oncology, Heidelberg, Germany; 3 Heidelberg Ion Therapy Center, Heidelberg, Germany; 4 Clinical Cooperation Unit Radiation Oncology, German Cancer Research Center (DKFZ), Heidelberg, Germany; 5 Department of Oral and Maxillofacial Surgery, University Hospital Heidelberg, Heidelberg, Germany; 6 National Center for Tumor Diseases (NCT), Heidelberg, Germany; 7 Department of Otorhinolaryngology, Head and Neck Surgery, University Hospital Heidelberg, Heidelberg, Germany; 8 Department of Radiation Oncology, University of Miami, Miami, FL, USA

**Keywords:** Head and neck cancer, local control, squamous cell carcinoma, extracapsular spread

## Abstract

**Background:**

Carcinomas of the lips are a relatively common malignancy of the head and neck region, accounting for roughly one quarter of all oral cavity cancers. Compared to other oral cancer sites, this location has a favorable prognosis, with 5-year survival rates between 85% and 95%. This study summarizes our institutional experience in utilizing postoperative radiation for patients with squamous cell carcinoma of the upper and/or lower lip following incomplete surgical resection or positive lymph node involvement with extracapsular extension.

**Methods:**

We retrospectively reviewed the medical records of all patients at the University Hospital of Heidelberg between 2005 and 2018 treated with postoperative radiotherapy of the upper and lower lip. Nineteen patients were identified with a median age at diagnosis of 67 years (range, 41–95 years), with 58% male and 42% female patients. Fourteen patients (73.7%) underwent neck dissection, with 5 (35.7%) found to have extracapsular extension (ECE) and positive resection margin (R1/2), 2 (14.3%) only ECE, and 7 (50.0%) with only R1/2. All patients received a median cumulative dose of 66.0 Gy (range, 60.0–70.0 Gy) in a median of 2.0 Gy per fraction (range, 1.8–2.2 Gy).

**Results:**

Median follow-up was 5.2 years. The median progression-free survival (PFS) was 3.9 years (range, 0.2–12.4 years), local disease-free survival (LDFS) was 4 years (range, 1–12 years) and overall survival (OS) was 5.2 years (range, 0.2–12.4 years). The 5-year Kaplan-Meier estimates for OS, PFS, and LDFS were 61.4%, 85.7%, and 100.0%, respectively. At last follow-up, 13 patients (68.4%) were still alive. Although no patient developed locoregional relapse, two patients developed distant relapse at a median of 15 months after radiotherapy. There was a statistically significant improvement in OS in patients treated with higher radiotherapy doses (>60.0 Gy, *p* = 0.044) compared to lower radiotherapy doses. PFS was significantly improved among patients who had N0 disease, with a negative resection margin, without ECE, and who were treated with intensity-modulated radiotherapy to doses >60.0 Gy. No grade 3/4 toxicity was detected; the most common grade 1/2 toxicities included dermatitis (n = 11, 57.9%), oral mucositis (n = 8, 42.1%), and dysphagia (n = 8, 42.1%).

**Conclusion:**

Our results demonstrate excellent local control and OS with acceptable toxicity when utilizing postoperative radiotherapy in patients with squamous cell carcinoma of the upper and lower lip, despite unfavorable characteristics (advanced T or N stage and/or ECE).

## Introduction

In Germany, 10,000 new cases of oral cancer are diagnosed every year.^1^ Histologically, over 95% of tumors in the oral cavity are squamous cell carcinomas (SCC).^2–4^ Carcinomas of the lip account for about one quarter of all oral cavity cancers. The most common involved site is the lower lip and occurs more often in male patients.^5,6^ In some geographic regions, the lips are the most common site of oral cancer. Incidence rates are around 13.5 per 100,000 in Oceania, 12 per 100,000 in Europe, and 12.7 per 100,000 in North America.^7–11^ The diagnosis of lip cancer often occurs early given the clinically apparent changes. Therefore, the majority of lip cancers are typically treated at an early stage with surgery and not often with radiotherapy (RT). Compared to other oral cancer sites, this location has a favorable prognosis.^12,13^

Surgery is the treatment of choice among lower T stages (T1/T2), whereas a combined approach is often utilized for advanced disease, with consideration for postoperative RT in the setting of positive resection margin (R1/R2) or extracapsular spread among involved lymph nodes to reduce the risk of local recurrence.^14,15^ Several studies have shown 5-year survival rates between 85% and 95% following postoperative RT^14–20^ (Table 1). Lymph node involvement is the most significant prognostic factor in this patient population,^5,21^ with studies showing that 5-year survival rates decrease to approximately 50% among those with N+ disease.^22^ The purpose of this retrospective study was to analyze local control rates and toxicity in patients with postoperative RT of SCC of the upper and lower lip who either had incomplete surgical resection or lymph node involvement. This study is also intended to update the data in the literature over recent years as well as to demonstrate our institutional experience.

**Table 1.** Overview of studies of postoperative radiotherapy in patients with squamous cell carcinoma of the upper and lower lip.

Authors (year)	Patients, n	Treatment time	RT technique	Median follow-up, mo	Treatment intention	LC at 5 years, %	OS at 5 years, %
Casal et al. (2010)^14^	29	1993–2000	3D CRT	62.1	R1, R2	87.2	Mortality rate 8.3
Najim et al. (2013)^15^	26	1980–2010	Orthovoltage energy photons (250–300 kV)	58.0	R1, R2, ECE	92	68
Fitzpatrick (1984)^20^	13	1971–1976	Photons (120 kV)	60.0	R1, R2, ECE	93	97
Veness et al. (2001)^19^	16	1980–1997	3D CRT	45.0	R1, R2, ECE	87.5	85
Current study	19	2005–2018	IMRT and 3D CRT	62.4	R1, R2, ECE	LDFS: 100; PFS: 85.7	61.5

CRT: conformal radiotherapy; ECE: extracapsular extension; IMRT: intensity-modulated radiotherapy; LC: local control; LDFS: local disease-free survival; OS: overall survival; PFS: progression-free survival; RT: radiotherapy.

## Methods

### Patient characteristics

Between 2005 and 2018, 78 patients were identified who were treated with surgery, chemotherapy, or RT for SCC of the lip at the University Hospital of Heidelberg. After excluding patients who were treated with either surgery alone, we retrospectively reviewed the records of 19 patients who were treated with postoperative RT in the Department of Radiation Oncology in Heidelberg. Only patients with histologically proven SCC were included in our analysis if they had either a positive resection margin (microscopic [R1]) or lymph node involvement with extracapsular extension (ECE). We excluded all patients with metastatic disease (M1) at initial diagnosis. Basic patient and treatment data were collected from the Heidelberg Nationales Centrum für Tumorerkrankungen (NCT) Cancer Registry. Clinical, operative, and hospital course records were reviewed. The median age was 67 years (range, 41–95 years), with 58% male and 42% female patients. The majority of patients presented with well-differentiated tumors with the most frequent location being the lower lip (12 patients, 63.2%) followed by 7 patients (36.8%) with upper lip cancer. There were 14 patients with microscopic positive resection margin (R1) and no patients with macroscopic resection margin. There were 9 patients (47.4%) treated with chemotherapy (cisplatin 40 mg/m² weekly). Information regarding a history of smoking was available for 71% of the patients, with the majority identifying as current or former smokers (73%). Detailed patient characteristics are shown in Table 2.

**Table 2.** Patient and treatment characteristics.

Characteristics	N (%) or median (range)
Sex	
Male	11 (57.9)
Female	8 (42.1)
Age, y	67 (41–95)
T stage	
T1	6 (31.6)
T2	8 (42.1)
T3	5 (26.3)
T4	0 (0.0)
N stage	
N0	7 (36.8)
N+	12 (63.2)
Resection margin	
R0	2 (14.3)
R1/2	17 (89.5)
Neck dissection	
Yes	14 (73.7)
No	5 (26.3)
Subgroups	
ECE positive + R1/2	5 (35.7)
ECE positive + R0	2 (14.3)
ECE negative + R1/2	7 (50.0)
Technique	
3D-CRT	4 (21.1)
IMRT	15 (78.9)
Total dose, Gy	66 (60–70)
Irradiation cervical lymph nodes	
Yes	18 (94.7)
No	1 (5.3)
Dose of cervical lymphatic drainage, Gy	54 (50–60)

CRT: conformal radiotherapy; ECE: extracapsular extension; IMRT: intensity-modulated radiotherapy.

### Treatment, follow-up, and toxicity

**Figure 1.**
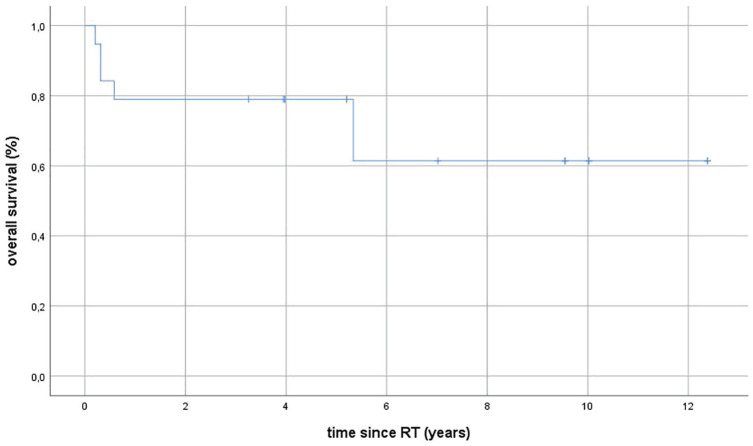
Kaplan-Meier estimates for overall survival (OS) in patients with squamous cell carcinoma of the upper and lower lips following postoperative radiotherapy (RT). The median OS was 5.2 years (range, 0.2–12.4 years).

All patients underwent surgical resection of the lip cancer with removal of all involved parts. In all patients, RT was carried out postoperatively using photon irradiation with either 3D-planned, image-guided intensity-modulated radiotherapy (IMRT) (TomoTherapy®; Accuray, Sunnyvale, CA) or volumetric modulated arc therapy (Elekta, Sweden), with treatment delivered one fraction per day and five fractions per week. Selection of the RT modality and dose fractionation was dependent on tumor characteristics, such as tumor thickness and lymph node involvement (Figure 1). Lip cancer is often stigmatizing; Figure 1 illustrates good cosmetic results 6 and 12 months after postoperative RT.

Aftercare for lip cancer consists of clinical examination. In our institution, follow-up consisted of computed tomographic (CT) imaging every 3 months within the first year after completion of RT, as well as regular clinical examination to evaluate outcome and potential tumor progression in the maxillofacial surgery department. After the first year, the frequency of CT imaging and clinical examinations was at 6-month intervals, and annually thereafter. Toxicity was classified according to the Common Terminology Criteria for Adverse Events v4.03 (CTCAE).

### Statistical analysis and outcome evaluation

Overall survival (OS), progression-free survival (PFS), and local disease-free survival (LDFS) were calculated using Kaplan-Meier analysis. OS was calculated as the time from start of RT until death or the date of last follow-up. PFS was calculated as the time from start of RT to tumor progression or death. LRFS was defined as the start of RT until local tumor progression at the primary tumor site. Patients still alive at the time of analysis, without tumor progression, or patients lost to follow-up were censored. Kaplan-Meier estimates were calculated using IBM SPSS software version 24. The results are presented as mean, range, and percentage. Subgroups were compared using the log-rank test. *p* Values of 0.05 or less were considered statistically significant. Odds ratios accompany 95% confidence intervals.

### Ethics

This study was performed following institutional guidelines and the Declaration of Helsinki of 1975 in its most recent version. Ethical approval for the study was given from the local ethics committee at University Hospital Heidelberg (S421-2015).

## Results

### Treatment results

All patients underwent a wedge resection with primary closure of the lip cancer with removal of all involved parts. Five patients required local flap reconstruction. Prophylactic neck dissection was not performed. All patients received upfront surgical resection followed by postoperative radiation treatment. Fourteen patients (73.7%) underwent neck dissection, either unilaterally (4 patients [28.6%]) or bilaterally (10 patients [71.4%]). Among the 14 patients with N+ disease, 5 patients (35.7%) had ECE and a positive resection margin (R1), 2 patients (14.3%) only ECE, and 7 patients (50.0%) only R1. In all patients, photon radiation was utilized once daily, five times per week, with one of the following techniques: 3D-conformal (21.0%), image-guided IMRT (78.9%). RT of the cervical lymph nodes was performed in 18 patients (94.7%). Twelve patients (63.2%) received concurrent systemic therapy. The median cumulative total dose was 66.0 Gy (range, 60.0–70.0 Gy) in a median of 2.0 Gy per fraction (range, 1.8–2.2 Gy/fraction). The median total dose to the lymph nodes was 54.0 Gy (range, 50.0–60 Gy). The main treatment features are listed in Table 1.

### Treatment outcome

The median follow-up was 5.2 years (range, 0.5–12.3 years). At the last follow-up, 13 patients (68.4%) were still alive. Among the six deaths, none were secondary to treatment-related toxicities: two caused by pulmonary infection, three by cardiac disease, and one of older age. Distant relapse was found in two patients (10.5%): one patient in lymph nodes outside of the treatment plan and the other in brain metastases. Distant relapse occurred at a median of 15 months after RT.

**Figure 2.**
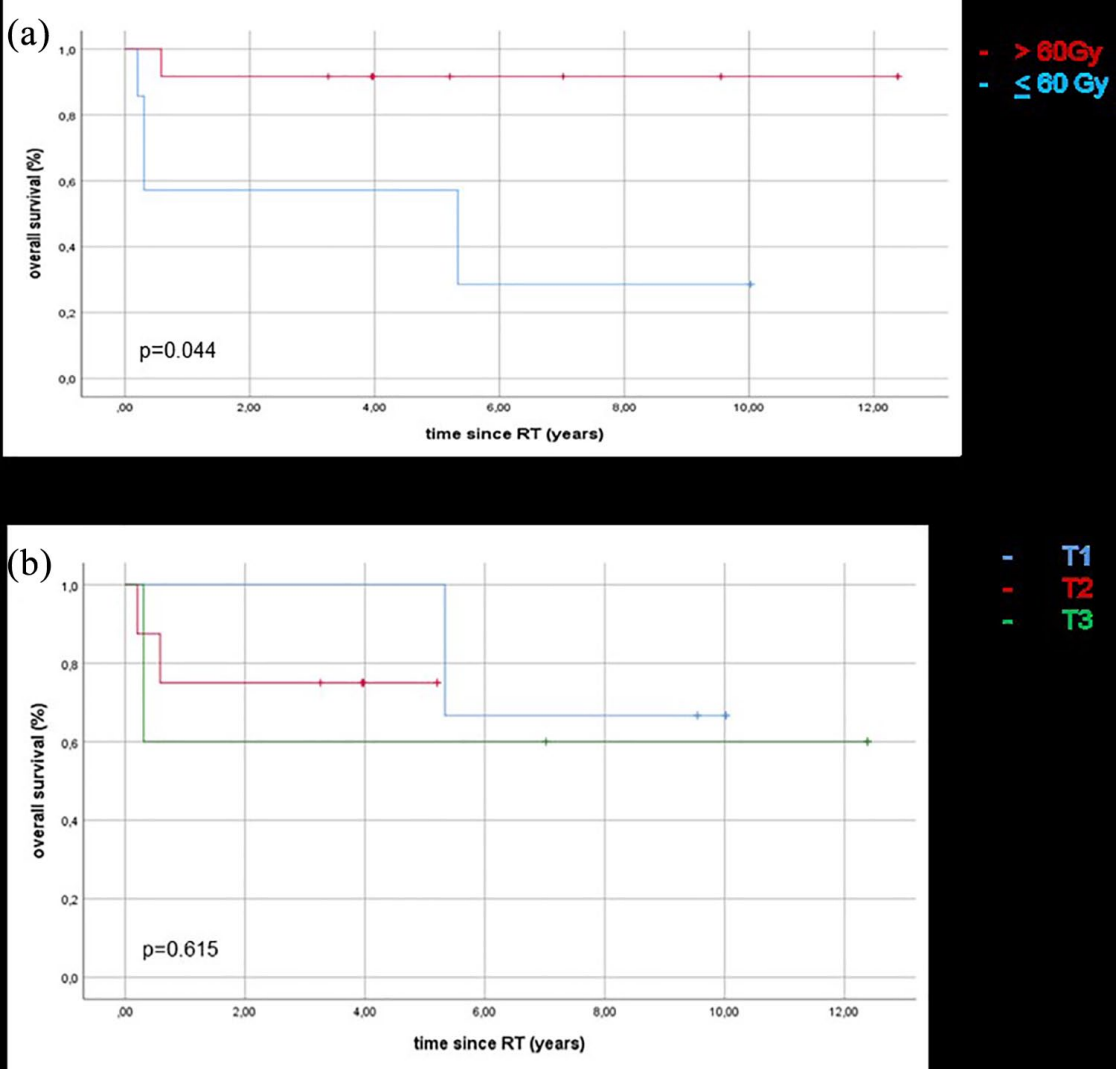
Kaplan-Meier estimates for overall survival (OS) (a) stratified by total dose (>60 Gy in red vs <60 Gy in blue), showing a significant improvement among those treated to a higher dose, and (b) stratified by T stage, showing no significant OS difference between the stages (p = 0.615).

Median PFS was 3.9 years (range, 0.2–12.4 years), LDFS 4 years (range, 1–12 years), metastasis-free survival 4 years (range, 0.2–12.4 years), and OS 5.2 years (range 0.2–12.4 years). The 2-year Kaplan-Meier estimates for OS, PFS, and LDFS were 78.9%, 85.7%, and 100.0%, respectively, and 5-year OS, PFS, and LDFS rates were 61.4%, 85.7%, and 100.0%, respectively. Kaplan-Meier estimates for OS of the entire cohort are shown in Figure 2.

### Univariate and multivariate analysis

**Figure 3.**
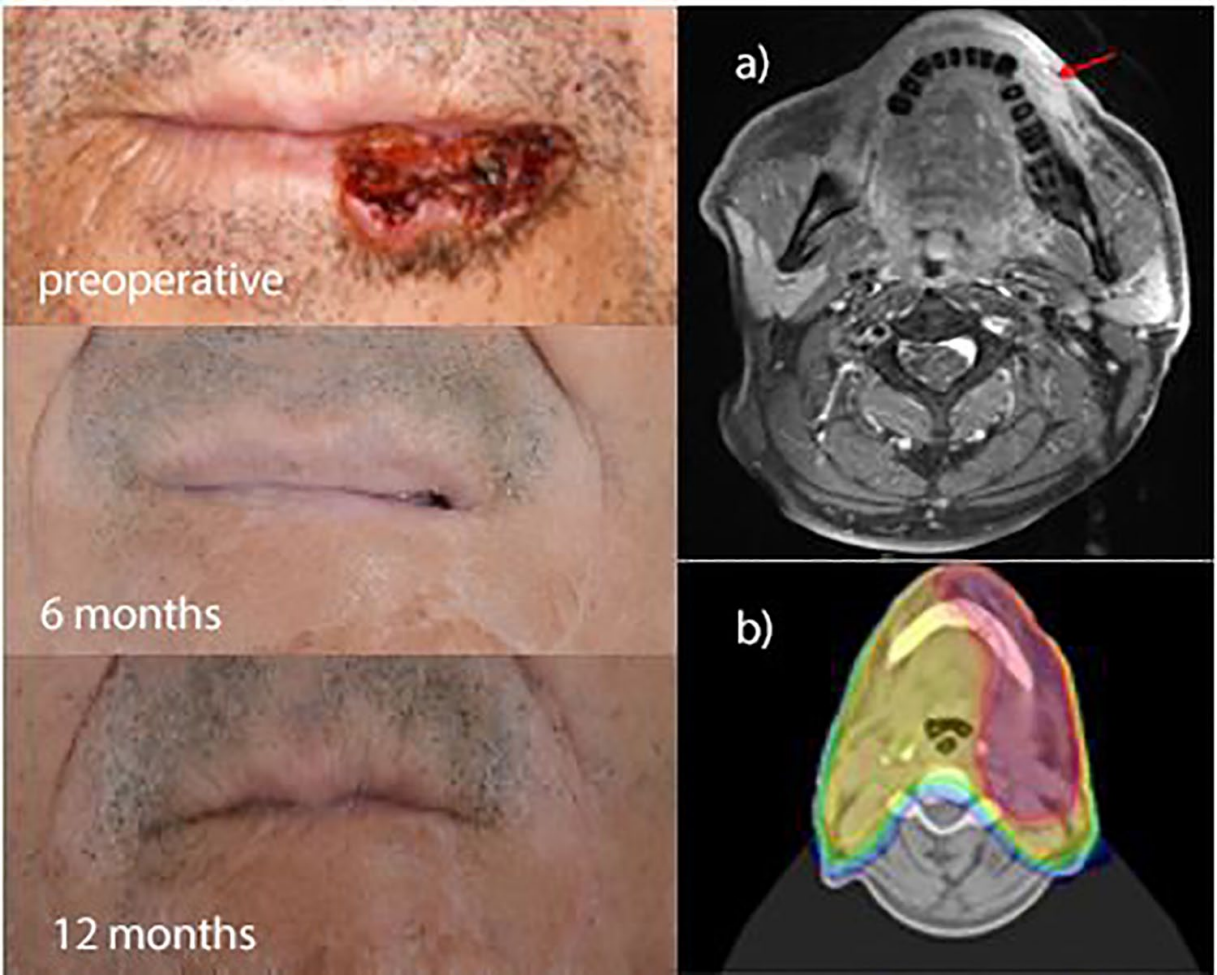
Upper left: 52-year-old man with squamous cell carcinoma of the lower left lip. The patient underwent radical surgical resection with staging identifying pN2b (5/35, extracapsular extension) G2, R0 disease, for which he was treated with postoperative radiotherapy to a cumulative total dose of 66 Gy to the lip and 50.4 Gy to the cervical lymphatic drainage. Middle left: 6 months after radiotherapy, showing no visible tumor. Lower left: 12 months after radiotherapy, showing evidence of recurrence. (a) Baseline transverse magnetic resonance imaging slice showing tumor extension to the alveolar bone without infiltration. (b) Intensity-modulated radiotherapy planning target volume (red) covering the 90% isodose.

The analysis showed a significantly improved OS in patients treated with RT doses >60 Gy (Figure 3, *p* = 0.04), without any other tumor or patient-related measure showing a significantly improved OS.

PFS was significantly improved among patients with N0 disease (*p* = 0.04), patients who had a negative resection margin (R0) (*p* = 0.04), patients who were treated with IMRT (*p* = 0.03), patients who had total dose >60 Gy (*p* = 0.026), and ECE-negative patients (*p* = 0.05). Among patients who were N+, those who were ECE-negative also had a statistically significant improvement in PFS (*p* = 0.046). There was no significant difference in OS, PFS, or LDFS in patients treated with concomitant chemotherapy (*p* = 0.34). Univariate analysis for OS and PFS are shown in Table 3. Multivariate analysis showed no significant predictor and association for OS and PFS.

**Table 3.** Univariate overall survival (OS) analysis of patient, tumor, and treatment-related factors.

Parameter	OS		PFS	
	HR (95% CI)	*p* Value	HR (95% CI)	*p* Value
Sex, male vs female	3.4 (0.6–18.5)	0.16	2.5 (0.15–40.0)	0.53
Age, y, ⩽70 vs >70	1.1 (0.9–1.1)	0.15	1.0 (0.06–16.0)	1.00
T stage, T1 vs T2/3/4	1.7 (0.3–10.2)	0.55	0.01 (0.00–11.9)	0.07
N stage, N0 vs N+	0.7 (0.1–3.5)	0.66	0.007 (0.00–17.3)^a^	0.04^a^
Location, upper vs lower	0.4 (0.04–3.4)	0.39	0.024 (0.00–41.1)	0.19
Neck dissection	0.5 (0.1–2.7)	0.40	25.5 (0.00–77.2)	0.44
ECE, negative vs positive	0.014 (0.0–44.3)	0.30	0.007 (0.00–16.2)^a^	0.05^a^
Resection margin, negative vs positive	1.2 (0.2–7.0)	0.83	0.007 (0.00–17.3)^a^	0.04^a^
RT technique, IMRT vs 3D	0.8 (0.1–4.8)	0.85	0.004 (0.00–77.5)^a^	0.03^a^
RT dose, Gy, >60 vs ⩽60	0.1 (0.01–0.95)	0.04	0.004 (0.00–77.5)^a^	0.03^a^

CI: confidence interval; ECE: extracapsular spread; HR: hazard ratio; IMRT: intensity-modulated radiotherapy; PFS: progression-free survival; RT: radiotherapy.

a Statistically significant findings.

### Toxicity

Treatment was well-tolerated without any severe treatment-related side effects.

The most common acute RT-related complications included grade 2 dermatitis (n = 11, 57.9%), oral mucositis (n = 8, 42.1%), dysphagia (n = 8, 42.1%), xerostomia (n = 3, 15.8%), edema (n = 2, 10.5%), and loss of taste (n = 4, 21.1%). There were no treatment-related deaths reported.

Supportive nutrition via a percutaneous gastric tube was required in one patient (5.3%) 10 days after the start of RT. The most common late RT-related complications (CTCAE grade 1–2) included xerostomia (n = 4, 21.1%), dermatitis (n = 7, 36.8%), and trismus (n = 2, 10.5%). Higher-grade late toxicity (CTCAE grade 3-4) was not detected. Treatment-related toxicities are summarized in table 4.

**Table 4.** Early and late toxicity after radiotherapy.

Early treatment toxicity (⩽90 days), CTC grade	N (%) of patients	Late treatment toxicity (>90 days), CTC grade	N (%) of patients
Mucositis		Dermatitis	
1	4 (21.1)	1	7 (36.8)
2	8 (42.1)	2	0 (0.0)
Dermatitis		Xerostomia	
1	6 (31.6)	1	3 (15.8)
2	11 (57.9)	2	1 (5.3)
Xerostomia		Trismus	2 (10.5)
1	13 (68.4)	Submental edema	1 (5.3)
2	3 (15.8)		
Dysphagia			
1	8 (42.1)		
2	8 (42.1)		
Loss of taste			
1	10 (52.6)		
2	4 (21.1)		
Fatigue	14 (73.7)		
Submental edema	2 (10.5)		

CTC: common terminology criteria.

## Discussion

We retrospectively reviewed our institutional outcomes in treating all patients with SCC of the lip treated with postoperative radiation. We found excellent local control and OS with acceptable treatment-related toxicity. Despite being grouped with other oral cavity cancers, lip carcinomas differ from other carcinomas of the oral cavity in terms of recurrent and metastatic spread. Lip carcinomas have a favorable prognosis in terms of OS and are one of the most curable tumors in the oral cavity.^23–26^ Yet there is a paucity of literature examining patients with poor prognostic features, which may not accurately reflect the true nature of this disease. Therein lies the rationale for the present study, to illuminate the outcomes of patients with advanced disease characteristics, including nodal disease spread and positive resection margin.

The median age in our study was 67 years (range, 41–95 years), with a slight male predominance (58% vs 42%), consistent with previously published reports noting a median age of 63–70 years with a higher incidence in male patients.^1,23,24^ In our cohort, only 19 of 78 patients with SCC of the lip required postoperative RT, with the remainder treated with surgery alone, the mainstay of treatment given the early disease presentation. An Australian study from Veness et al. reported that only a small number of patients required postoperative treatment with RT or chemotherapy (or both), similar to our observations.^19^ In the present series, 14 patients had early stage disease (T1/2), with ECE lymph node metastases identified in 36.8% of patients.

Several studies reported a recurrence-free survival of 86.1% at 10 years with postoperative RT.^27,28^ These findings are similar to those presented in the current study, with 5-year PFS and LDFS rates of 85.7% and 100.0%, respectively; additionally, no patient developed locoregional relapse. Two patients, however, both with a positive resection margin (R1) and extracapsular spread, developed recurrent disease with distant metastasis. In previously published reports, T stage was identified as a prognostic factor for both local recurrence and OS.^22,26^ Lesion size was not a predictor of worse OS and PFS on univariate analysis. This distinction tends to lose significance in light of recent surgical advancements over the past several decades, whereby complete excision with negative excision margins has become standard of care. Whereas we failed to show that margin status predicted a worse prognosis, Zitsch et al.^22^ reported worse survival in patients with involved margins compared to patients with clear margins (*p* < 0.024). In our study, 5 patients had a complete excision with a median margin of 2 mm (range, 1–7 mm). The definition of an acceptable surgical margin is not well defined, yet standard practice identifies a margin of 2–10 mm for the best outcome. Brodland and Zitelli^29^ described that tumors with a diameter greater than 2 cm require a resection margin up to 6 mm to achieve a good response with a lower rate of local recurrence. Our study had too few cases to illuminate what role the extent of margin status has on outcome. Published data recommending postoperative RT for patients with close or positive margins or among patients who have extracapsular spread in involved lymph nodes, with the aim of reducing risk of local recurrence, are lacking in literature. There are published studies recommending postoperative RT, but the reasons are missing.^22^ Veness et al.^19^ showed improved survival when using postoperative RT in patients with risk factors, including insufficient resection margin, which could also explain our data. Despite risk factors including ECE and R1/2 resection, local control was 100%. In our study, two patients (10.6%) died as a result of distant relapse of their lip carcinoma (one patient with cutaneous metastasis [submental] and the other with distant positive lymph nodes out of field) while receiving systemic therapy with palliative intent. Other published studies, including Zitsch et al.^22^ and Grover et al.,^30^ reported similar results to ours, with a cancer-related mortality of 7%.

In terms of OS, our results appear consistent with historical trends: Warnakulasuriya^25^ identified that over 90% of patients survive for 5 years after first diagnosis, and we observed 2- and 5-year OS rates of 100%.

Given the intricate role that the lips play in our sense of being human as well as routine oral cavity functioning, any treatment-related toxicity plays a crucial role in quality of life,^31,32^ underscoring the significance of examining acute and long-term side effects.

RT-induced damage to the oral mucosa was observed, as well as damage to the skin, salivary glands, and masticatory apparatus, but the rates of both early and late toxicity are comparable with other published series.^18,20,33,34^ The incidence of acute toxicities in our study was very low, with the most common acute RT-related complications (CTCAE grade 1–2) including dermatitis (n = 11, 57.9%), oral mucositis (n = 8, 42.1%), dysphagia (n = 8, 42.1%), xerostomia (n = 3, 15.8%), and loss of taste (n = 4, 21.1%). Our most notable long-term toxicity was swallowing dysfunction (21.1%) and trismus (10.5%), comparable to results found in the literature.^18,20,33,34^

This study has several limitations, predominately related to its retrospective nature and the relatively small number of patients. For example, toxicity data were limited by medical documentation. Yet it is worth highlighting the length of the analysis (spanning 13 years) and the general paucity of patients requiring adjuvant treatment for what is often an early-stage disease. Among the strengths of the present analysis, all patients were treated by a consistent group of radiation, surgical, and medical oncologists at a single institution. We also examined a cohort with more advanced characteristics, which may more accurately reflect the true nature of this disease.

Finally, although this work is a retrospective analysis with a small number of patients, the power of this study is that we analyzed a well-selected collective with a complete and long follow-up at a large department with a lot of experience in tumor diseases. All follow-up CT scans were reviewed by an experienced radiologist by institutions’ own diagnostics.

Our study confirmed that postoperative IMRT for patients after nonradical lip cancer surgery is an efficient and safe treatment option. It is in agreement with other retrospective studies; however, as shown in Table 1, data with large patient collectives are not available. Our analysis reflects the most recent data. Postoperative RT for patients with SCC of the lips should be offered due to good tumor control with few side effects.

## Conclusion

Postoperative RT in patients with SCC of the upper and lower lip for either incomplete excision or positive extracapsular spread in lymph nodes is associated with good local and distant control as well as overall survival rates. Patients should be treated with cumulative doses higher than 60 Gy to the primary tumor site for improved outcomes.
